# Differential effect of quetiapine and lithium on functional connectivity of the striatum in first episode mania

**DOI:** 10.1038/s41398-018-0108-8

**Published:** 2018-03-06

**Authors:** Orwa Dandash, Murat Yücel, Rothanthi Daglas, Christos Pantelis, Patrick McGorry, Michael Berk, Alex Fornito

**Affiliations:** 1 Brain & Mental Health Laboratory, School of Psychological Sciences & Monash Institute of Cognitive and Clinical Neurosciences, Monash University, Clayton, VIC 3168 Australia; 2 Melbourne Neuropsychiatry Centre, Department of Psychiatry, The University of Melbourne and Melbourne Health, Carlton South, VIC Australia; 3 Orygen, The National Centre of Excellence in Youth Mental Health, 35 Poplar Road, Parkville, VIC 3052 Australia; 4 Centre for Youth Mental Health, University of Melbourne, 35 Poplar Road, Parkville, VIC 3052 Australia; 5 Florey Institute for Neuroscience and Mental Health, Kenneth Myer Building, Royal Parade, Parkville, VIC Australia; 6 IMPACT Strategic Research Centre, School of Medicine, Deakin University, Geelong, VIC 3220 Australia; 7 Orygen Youth Health Clinical Program, 35 Poplar Road, Parkville, VIC 3052 Australia; 8 Barwon Health and the Geelong Clinic, Swanston Centre, Geelong, VIC 3220 Australia

## Abstract

Mood disturbances seen in first-episode mania (FEM) are linked to disturbed functional connectivity of the striatum. Lithium and quetiapine are effective treatments for mania but their neurobiological effects remain largely unknown. We conducted a single-blinded randomized controlled maintenance trial in 61 FEM patients and 30 healthy controls. Patients were stabilized for a minimum of 2 weeks on lithium plus quetiapine then randomly assigned to either lithium (serum level 0.6 mmol/L) or quetiapine (dosed up to 800 mg/day) treatment for 12 months. Resting-state fMRI was acquired at baseline, 3 months (patient only) and 12 months. The effects of treatment group, time and their interaction, on striatal functional connectivity were assessed using voxel-wise general linear modelling. At baseline, FEM patients showed reduced connectivity in the dorsal (*p* = 0.05) and caudal (*p* = 0.008) cortico-striatal systems when compared to healthy controls at baseline. FEM patients also showed increased connectivity in a circuit linking the ventral striatum with the medial orbitofrontal cortex, cerebellum and thalamus (*p* = 0.02). Longitudinally, we found a significant interaction between time and treatment group, such that lithium was more rapid, compared to quetiapine, in normalizing abnormally increased functional connectivity, as assessed at 3-month and 12-month follow-ups. The results suggest that FEM is associated with reduced connectivity in dorsal and caudal corticostriatal systems, as well as increased functional connectivity of ventral striatal systems. Lithium appears to act more rapidly than quetiapine in normalizing hyperconnectivity of the ventral striatum with the cerebellum. The study was registered on the Australian and New Zealand Clinical Trials Registry (ACTRN12607000639426). http://www.anzctr.org.au

## Introduction

First-episode mania (FEM) patients can exhibit difficulties in emotion regulation and deficits in cognitive function such as attention, response inhibition and working memory^1^ that are commonly linked to the functional integrity of neural circuits connecting prefrontal cortex (PFC) with subcortical and limbic structures^2–5^.

Key among these are the cortico-striato-thalamic (CST) and cortico-striato-cerebellar (CSC) networks, which are thought to mediate a broad array of cognitive, motor and affective processes^6,7^. The striatum is as an important structure in these networks, representing a key point for integrating diverse cortical and cerebellar inputs. Broadly, the functional organization of the striatum evolves along dorsal-to-ventral and medial-to-lateral axes which separate the associative striatum (head of the caudate nucleus and dorsorostral putamen) from the motor (caudate tail and dorsocaudal putamen) and the limbic (ventral striatum/nucleus accumbens) subdivisions^6,8^. The striatum is thus well-positioned to play a critical role in functions that integrate cognitive processes with emotional drives to implement motor outputs—an ability that is thought to be fundamentally disrupted in bipolar disorder^9^.

A popular method for studying striatal and other brain networks is to use functional magnetic resonance imaging (fMRI) to record coordinated, spontaneous brain dynamics in the absence of an explicit task (the so-called resting-state)^10,11^. The signal fluctuations recorded with resting-state fMRI are organized into well-defined networks^12^, including specific CST and CSC systems^13–15^. These networks are robust over time^16,17^, heritable^18^ and influence task-evoked activity and behavior^19,20^, suggesting that they represent an intrinsic and functionally important property of brain activity^21^.

Several resting-state fMRI studies have found that patients with bipolar disorder show disturbances in coordinated brain activity—so-called functional connectivity—between the striatum and other brain areas^22–24^. In the largest resting-state study to date, increased connectivity between a medial paralimbic resting-state network, involving the ventromedial prefrontal cortex and the ventral striatum, with a network thought to subserve emotion regulation and executive function, including subgenual cingulate and insula, distinguished bipolar patients from schizophrenia patients and healthy controls, suggesting that interactions between cognitive and emotional networks may be specifically disrupted in bipolar disorder^24^. Similarly, another analysis found that functional connectivity between the basal ganglia, thalamus and cortical areas implicated in cognition were the most distinguishing feature between bipolar patients and healthy controls, achieving a classification accuracy of 90%^25^. Furthermore, altered functional connectivity in the cognitive and affective subdivisions of the striatum has been shown to differentiate manic patients from depressed bipolar patients^26^. Collectively, these findings suggest that bipolar disorder is associated with abnormal coupling between striatal and other brain areas involved in cognitive and emotional processes.

Most fMRI studies have included patients treated with the mood stabilizer lithium, second-generation antipsychotics such as quetiapine, or adjunctive therapy^22–24^. The evidence from randomized-controlled studies suggests that quetiapine is an effective treatment of bipolar mania^27^ and bipolar depression^28^, for maintenance therapy^29^, and as an adjunct treatment to lithium, where efficacy may be greater than quetiapine monotherapy^30^. However, no study to date has investigated the differential effect of monotherapy of either of the drugs on resting-state functional connectivity in First Episode Mania (FEM) patients. This is mainly due to the low incidence of FEM and the consequent difficulty in recruiting affected patients^31,32^. In addition, previous studies have used cross-sectional designs, which do not allow an assessment of how treatment changes brain function over time. More importantly, there are no randomized designs in these cohorts.

In this study, we aimed to investigate changes in the resting-state functional connectivity of four key regions of the striatum, encompassing dorsal (cognitive), ventral (affective) and caudal (motor) circuits in FEM patients randomized to either quetiapine or lithium. Patients were scanned at baseline, 3 months and 12 months, with a sample of healthy controls also scanned at the baseline and 12-month assessments. We expected that functional connectivity of both the dorsal and ventral striatum would be altered in patients compared to controls at baseline. We also predicted, in light of the evidence for the clinical efficacy of both lithium and quetiapine in treating manic patients^27,29,30^, that treatment with both drugs would normalize baseline abnormalities over the follow-up period.

## Subjects and methods

### Participants

Sixty-one participants with FEM were recruited via Orygen Youth Health and Monash Health between December 2008 and December 2013. Patients were screened with the Structured Clinical Interview for DSM-IV-TR by trained clinicians and were diagnosed with mania (bipolar I disorder, schizoaffective disorder, or a substance-induced mood disorder). Individuals presenting with an acute manic episode with psychotic features and who had not been previously treated for a manic episode were stabilised on a combination of quetiapine plus lithium in an open label manner as part of a routine care protocol. Following provision of informed oral and written consent, patients were randomised after remission (2–3 months), to lithium or quetiapine monotherapy. Patients were required to have been on a combination of quetiapine and lithium as standard therapy for at least 1 month prior to randomisation, and to have a score of at least 20 on the Young Mania Rating Scale (YMRS)^33^ during their acute manic episode. Female patients were required to be using effective contraception if they were sexually active and of childbearing age. Patients of 15–25 years of age, who were fluent in English, had the capacity to provide informed consent, and comply with study procedures, were randomised to treatment allocation of either lithium or quetiapine monotherapy. The Melbourne Health and Monash Health Human Research Ethics Committees approved the protocol in accordance with the Helsinki Declaration.

### Exclusion criteria

Patients were excluded for known or suspected clinically relevant systemic medical disorder, organic mental disease or a history of epilepsy; sensitivity or allergy to quetiapine, lithium or any additives in the medication; prior use of medication with a cytochrome P450 3A4 inhibiting effect in the 14 days preceding enrolment; inability to comply with the requirements of giving informed consent or the treatment protocol; immediate risk of self-harm or of harming others; pregnancy or breastfeeding; or diabetes mellitus.

### Randomisation and blinding

At the discretion of the treating team an independent statistician generated a computerised randomisation sequence 2–3 months following stabilisation from a FEM. A randomisation log was established and a set of sequentially ordered envelopes was kept in a locked filing cabinet at the Orygen Research Centre. The patients, treating psychiatrist and case managers knew which treatment the patient was receiving while research assistants, neuropsychologists, and all individuals involved in neuroimaging, analysis, and data management remained blinded to this information.

### Study protocol

Of the 61 recruited subjects, 31 subjects were allocated to quetiapine treatment and 30 patients were allocated to lithium treatment. A single researcher (RD) conducted all clinical and diagnostic assessments. Clinical assessments were carried out at baseline and on fortnightly intervals for the first month, then on a monthly basis for the following 2 months, and then at 3 monthly intervals thereafter concluding at the 1-month time point. These included observer-based ratings using the YMRS^33^ for manic symptoms, the Montgomery-Åsberg Depression Rating Scale MADRS;^34^ for depressive symptoms, the Brief Psychiatric Rating Scale BPRS;^35^; for psychotic symptoms, and the Clinical Global Impressions scale for use in Bipolar disorder CGI-BP;^36^; to determine overall symptom severity. Control subjects underwent MRI scanning at baseline and at the 12-month time points whereas FEM patients were scanned at baseline, 3 and 12-month time points to assess response to treatment.

### MRI data acquisition

A single scanner at the Murdoch Children’s Research Institute at Royal Children’s Hospital in Melbourne, Australia (3 T Siemens Trio Tim, 32 channel head coil) was used to acquire high-resolution structural T1 Magnetization-Prepared RApid Gradient-Echo (MPRAGE)^37^ scans for each subject. Image acquisition parameters at every time-point were as follows: 192 sagittal slices with a nominal 1mm^3^ voxel size, 256 mm × 232 mm Field-of-View (FoV) and a matrix size of 256 × 192 or 512 × 384 pixel resolution (the latter had 0.5 × 0.5 mm in-plane resolution), 2000 ms repetition time (TR) and 2.24 ms echo time (TE). T2*-weighted echo-planar images were acquired under eyes-closed resting-state conditions. Subsequent participant debriefing ensured that no participants fell asleep during the scan. Functional MRI was acquired using a T2*-weighted sequence with 3.3 × 3.3 × 3.0 mm voxel size, a TR of 2400 ms, TE of 30 ms, flip angle of 90° in 64 × 64 matrix size and 384 mm FoV. A total of 307 volumes comprising 36 slices each were acquired.

### Imaging preprocessing

An established procedure was used to characterize corticostriatal functional connectivity in relation to four seed regions located in ventral and dorsal areas of the caudate nucleus and putamen per hemisphere^13,38,39^. Seeds were defined in both hemispheres as 3.5 mm radial spheres at the following stereotaxic coordinates: dorsal caudate (*x* = ±13, *y* = 15, *z* = 9); ventral striatum/nucleus accumbens (*x* = ±9, *y* = 9, *z* = −8); dorsal-caudal putamen (*x* = ±28, *y* = 1, *z* = 3) ventral-rostral putamen (*x* = ± 20, *y* = 12, *z* = −3). A secondary seed-based analysis was conducted in which two 3.5 mm spherical seeds were placed in the right and left visual cortex as described elsewhere^40^. Image preprocessing was performed using statistical parametric mapping software (SPM8) and included slice-timing correction, motion correction via affine transformation to the first image and coregistration of functional images with subjects’ anatomical scans, which were concurrently normalized to the SPM-T1 template. The resulting transformation matrix was applied to the functional data to achieve accurate spatial normalization across individuals. The anatomical scans were also segmented using a unified normalization and segmentation approach^41^.

White matter and CSF were generated by thresholding the corresponding tissue images segmented from the T1 scan at 99 and 50% tissue probability, respectively. Any overlap with the grey matter mask (thresholded at 1%) was removed by image subtraction. Voxelwise time series were extracted from the white matter and CSF masks and subjected to the aCompCor method^42^, in which voxelwise time course from each tissue mask were subjected to separate principal component analyses. The first five components from each mask were retained as nuisance regressors modelling physiological noise and head motion effects. All data were linearly detrended and a linear regression model that included these ten component signals, together with the six head motion parameters (three rotation, three translation) estimated during the head motion correction procedure, and the first-order derivatives of all sixteen signals were then fitted on a voxelwise basis. This approach has been shown to successfully control for head motion and physiological noise in fMRI data^43^. The noise-corrected data were then band pass filtered (0.008 < *f* < 0.08) and spatially smoothed with a Gaussian filter (8 mm full-width at half-maximum). All image sequences were routinely inspected for potential normalization artifacts. Mean time series were extracted from each striatal seed in both hemispheres. The data of one patient at baseline was excluded due to excessive head motion (>3 mm translation and >3° rotation). This resulted in a final sample of 30 control subjects, and 40 patients at baseline. The final sample for both treatment groups at 3 months (denoted m3) was 13 subjects in the quetiapine group and 16 subjects in the lithium group. At 12 months (denoted m12), there was 20 subjects in the control group, 12 subjects in the quetiapine group and 14 subjects in the lithium group.

### First-level, within-subjects analysis

For each participant, functional connectivity maps were estimated using general linear models (GLM) as implemented in SPM8. Time courses extracted from each striatal sub-region were entered into a single GLM that included all striatal seeds as covariates of interest in a whole-brain regression analysis. Contrast images were generated for each participant by estimating the regression coefficient between all brain voxels and each region’s time-series for left and right hemisphere seeds.

### Second-level, between-condition analysis

Separate second-level models were used to test for (1) baseline disease-related changes in corticostriatal effects, and (2) the longitudinal effects of treatment. For the first analysis, separate models were used to test for group differences in the functional connectivity of each of the four seed regions, with group (patient, control) and hemisphere (left and right) as covariates of interest and age and gender as nuisance covariates. Between-group statistical maps were thresholded at a *P* < .001 uncorrected and subjected to cluster-based correction for multiple comparison at *P* < .05 determined by a Monte Carlo simulation test^44^ based on the recently amended 3dClustSim algorithm^45^.

The longitudinal effect of treatment was characterized using a 3 × 2 random-effects flexible factorial design with group (control, quetiapine and lithium) and time (baseline, m12) as factors and age and gender as nuisance covariates. A separate 2 × 3 random-effect design was used to characterize the effect of treatment at m3 in the patients groups only. Longitudinal effects were mapped by masking the *F*-contrast for the group × time interaction with the statistical map of the baseline differences between patients and controls to ensure that the influence of treatment was characterized in brain regions that are (1) functionally connected to the striatum; and (2) of primary pathophysiological relevance to the disorder. Interaction effects within this mask that survived a threshold of *P* < .05 small volume-corrected, were deemed significant.

### Clinical associations

Change from baseline scores were computed for the BPRS total (ΔBPRStot12), MADRS (ΔMADRS12), CGI-BP severity of depression (ΔCGI-BPsevDepres12), and CGI-BP overall severity of bipolar (ΔCGI-BPsevBP12) by subtracting m12 clinical scores from baseline scores^46^.

## Results

### Demographics and clinical outcomes

**Fig. 1:**
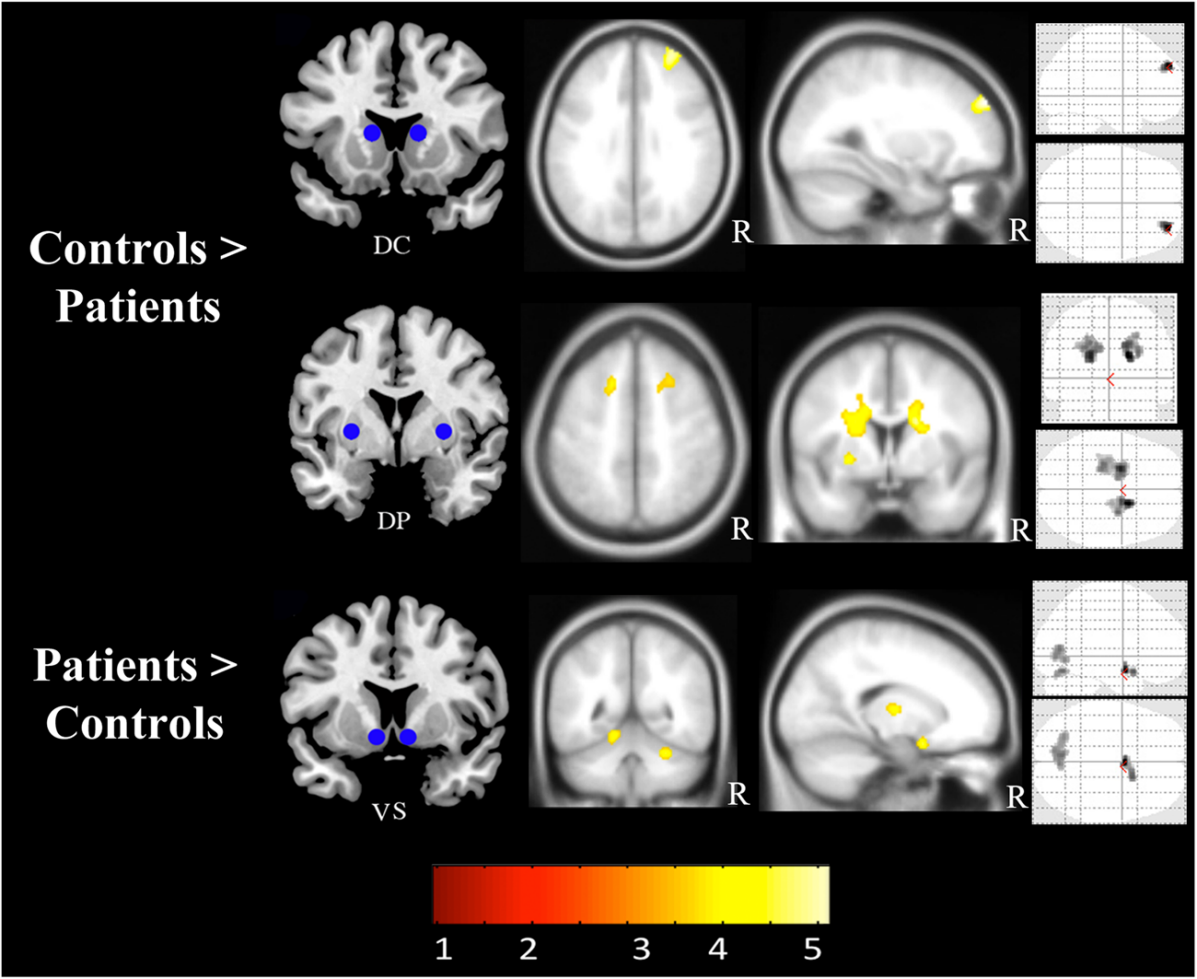
*Z*-score statistical map of significant baseline comparison in functional connectivity between control subjects and first-episode mania patients collapsed across both treatment groups; quetiapine and lithium. DC dorsal caudate nucleus, DP dorsal putamen, VS ventral striatum including the nucleus accumbens. R right hemisphere. Insets show results overlaid on glass brain. Results are displayed at *P* < 0.05 (FWE cluster corrected). For more information see Supplementary Materials

Of the 61 recruited subjects, 11 in the quetiapine treatment group and 9 in the lithium treatment group were excluded at or before randomization took place for the following reasons; relapse at baseline or prior to commencement of monotherapy (*n* = 5); self-ceasing of all medications (*n* = 3); non-stability on monotherapy (*n* = 2); preference for the non-randomized medication (*n* = 3); clinician withdrawal due to side effects (*n* = 2) and treatment disengagement (*n* = 4) (Fig. 1). Twenty-one patients were allocated to quetiapine treatment and 20 subjects were allocated to lithium. Two additional subjects were excluded at baseline; one subject due to never being on monotherapy and one subject due to non-compliance to randomized medication (see CONSORT flowchart in Supplement for more information) rendering a final sample of 19 subjects in the quetiapine group and 20 subjects in the lithium group. Three patients in the quetiapine group and four in the lithium group discontinued after their baseline participation.

Treatment groups were matched for age, gender, handedness and IQ but only matched for age and handedness with the control group (Table 1). Baseline clinical scores did not differ between the two treatment groups (patients assigned to lithium or quetiapine), indicating that stabilization was successful. As reported in refs 46,47, the lithium group demonstrated significant improvement compared to quetiapine after 12 months on rating scales of depression, psychosis, and mood instability.

**Table 1:** Demographics of treatment groups and control subjects

Demographics	Control *N* = 30		Quetiapine (*N* = 19)		Lithium (*N* = 20)		Statistics	
	*N*	%	*N*	%	*N*	%	*χ* ^*2*^	*P*
Gender								
Male	12	40	14	70	16	84	9.87	.007
Female	18	60	5	30	4	16		
*Handedness* ^a^								
Right-handed	26	93	15	83	19	95	3.64	.162
Left-handed	2	7	3	17	0	0		
Age	Mean	s.d.	Mean	s.d.	Mean	s.d.	*F*	*P*
	21.40	2.46	21.47	2.14	21.45	2.31	0.145	.88
Premorbid IQ (WTAR)^b^	105.43	10.82	92.89	13.80	96.71	13.89	11.37	.001
Diagnosis								
Bipolar disorder	—	—	19	100	17	85	1.89	1.17
Schizoaffective disorder	—	—	1	5	1	5	0.04	1.84
Substance-induced Mania	—	—	1	5	1	5	0.08	1.80

^a^Data for two subjects in the control group and one subject in the quetiapine and the lithium group were missing.

^b^WTAR: Wechsler Test of Adult Reading (UK scaled score)

### Baseline effects of disease

At baseline, all patients (i.e., both treatment groups), when compared to controls, demonstrated increased functional connectivity in the putative affective corticostriatal network, linking the ventral striatum (nucleus accumbens) and the ventromedial orbitofrontal cortex (vmOFC), cerebellum, and the thalamus in the right hemisphere (Fig. 1a). Patients showed reduced functional connectivity between the dorsal caudate and DLPFC in the right hemisphere (Fig. 1; Table 2). Patients also showed reduced connectivity between the dorsocaudal putamen and the premotor area, and between the same putamen seed and the caudate nucleus bilaterally (Fig. 1; Table 2). There was no significant difference in functional connectivity between the two treatment groups at baseline.

**Table 2:** Brain regions demonstrating significant between-group differences in functional connectivity (*P* < 0.05; FWE cluster corrected) and group × time interactions (*P* < 0.05; small-volume corrected)

Main Effect	Anatomical Region	Hemisphere	MNI Peak Coordinates (*x*,*y*,*z*)	*Z*-score	Voxels
DC	DLPFC	Right	30,54,34	4.16	156
PTdc	Caudate Nucleus	Right	18,6,22	4.53	632
	Putamen Globus Pallidus	Left	−26,−2,−2	4.28	167
	Caudate Nucleus	Left	−22,−2,20	4.27	1043
	Premotor Cortex	Left	−18,12,56	3.56	183
	Premotor Cortex	Right	24,14,46	3.52	120
VS	mOFC	Right	4,0,−18	4.59	225
	Thalamus	Right	16,−12,6	3.94	140
	Cerebellum	Left	−14,−48,−14	3.93	120
	Cerebellum	Right	28,−50,−28	3.91	134
Group x Time Interaction					
VS	Cerebellum	Right	32,−48,−28	3.73	73

*DC* dorsal caudate, *PTdc* dorsocaudal Putamen, *VS* ventral striatum.

### Longitudinal effects of treatment

**Fig. 2:**
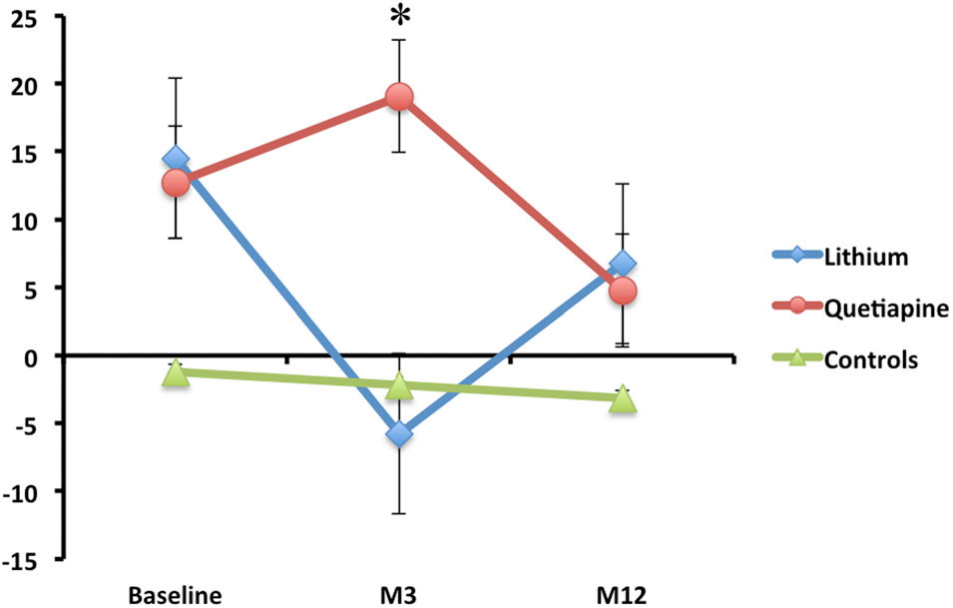
Group × time interaction in ventral striatum (VS) functional connectivity with the Right cerebellum. *Significant at *P* < .05 small volume corrected. Error bars represent standard error of the mean. See Results section for more information on between group comparisons

A significant group × time interaction was identified for functional connectivity between the ventral striatum and right cerebellum (Fig. 2 and Table 2). Post-hoc *t*-tests showed that both treatment groups showed increased connectivity when compared to control subjects at baseline (quetiapine *t*_1,68_ = 3.0, *P* = .001 cluster-corrected; lithium *t*_1,68_ = 3.6, *P* = .001 cluster-corrected). At m3, patients treated with lithium showed reduced functional connectivity compared to those treated with quetiapine (*t*_1,27_ = 2.0, *P* < .05 svc). After m12, the two treatment groups did not differ in their connectivity strength, although both were significantly higher than controls (Fig. 2; quetiapine *t*_1,30_ = 1.6, *P* < .05 svc; lithium *t*_1,34_ = 1.75, *P* < .05 svc).

### Clinical associations

In an exploratory analysis, we next examined whether the treatment-related changes in functional connectivity were related to symptom improvement. We extracted estimate of functional connectivity from the cerebellar region showing a significant group × time interaction and correlated these estimates with measures of symptom change. In the lithium group, functional connectivity between the ventral striatum and cerebellum was associated with symptom improvement on the following scales in the lithium group: ΔCGI-BPsevDepres12 (*r* = 0.51, *n* = 14, *P* = .022) and ΔCGI-BPsevBP12 (*r* = 0.51, *n* = 14, *P* = .021). However, none of these associations survived full Bonferroni correction for multiple comparisons (*α* = 0.0125).

An additional exploratory association analysis was conducted in which baseline functional connectivity estimates, outside of the brain regions that represent between-group differences (Fig. 1), were examined to see whether they predict symptom improvement after 12 months. Symptom scores were entered as variables of interest in a voxel-wise whole-brain regression analysis while controlling for age and gender. Baseline functional connectivity predicted improvement in symptom scores in both treatment groups after 12 months (Supplementary Figures 2 and 3). Specifically, connectivity of the ventral striatum with the right temporal cortex predicted improvement on BPRS (*r* = 0.83, *n* = 14, *P* = .012 FWE Cluster corrected) while connectivity between the ventral striatum and the right middle frontal gyrus predicted improvement on MADRS scores (*r* = 0.86, *n* = 14, *P* = .001 FWE Cluster corrected) in the lithium group (Supplementary Figure 1). In the quetiapine group, connectivity of the ventral striatum and the ACC predicted improvement on BPRS scores (*r* = 0.68, *n* = 12, *P* = .001 FWE Cluster corrected) (Supplementary Figure 2). These effects survived full Bonferroni correction for multiple comparisons (*α* = 0.0125).

There was no clinical association with the connectivity of the visual cortex as suggested by the secondary analysis (Supplementary Figure 1). This latter analysis supports the specificity of the results to ventral striatum connectivity.

## Discussion

FEM patients showed reduced functional connectivity of dorsal cortico-striatal circuitry, coupled with abnormally increased functional connectivity of a ventral CSC network. The connectivity abnormalities normalized to a similar extent over the 12-month period of this study in patients treated with either lithium or quetiapine, but the changes occurred earlier in the lithium-treated group, being apparent at 3 months. These findings suggest that the therapeutic effect of quetiapine in FEM may be delayed relative to lithium, at least with respect to normalizing functional connectivity of the ventral striatum with the cerebellum. We note however, that the abnormalities did not completely normalize, with residual differences between the two patient groups and healthy controls still apparent at 12 months.

Paralleling its traditional role in controlling movement, the cerebellum plays an important role in modulating emotions^48,49^. The cerebellum sends monosynaptic projections to the basal ganglia as well as other limbic subcortical structures including the hippocampus and the amygdala^7,48^. Lesions to the posterior and vermal cerebellum lead to disinhibition and inappropriate behavior, a characteristic feature of mania^50^. Moreover, the cerebellum commonly appears as one of the most activated brain regions during emotion appraisal^51,52^ and a reduction in cerebellar volume is associated with the number of affective episodes, including manic and hypomanic episodes, in bipolar patients^53^.

In a recent volumetric study of this cohort, we found reduced cerebellar volume in FEM patients at baseline, when compared to control subjects, in the same region that shows increased connectivity with the ventral striatum^54^. These findings further support the role of the cerebellum in mediating affect and suggest that increased functional connectivity in the limbic circuit may be related to the structural deficit in the cerebellum. One hypothesis is that increased functional connectivity with the ventral striatum represents a compensatory response to a structural lesion in this area, although testing this hypothesis would require a thorough behavioural assessment^55^. Unlike our previous study, which found that cerebellar volume was not responsive to treatment^54^, we report here that medication improves striatocerebellar connectivity, suggesting that functional measures may be more sensitive to the effects of pharmacotherapy (Fig. 2).

Changes in functional connectivity of the ventral striatum were accompanied by improvement in symptoms after 12 months in the lithium group only. This finding was further cemented by the secondary exploratory analysis in which baseline functional connectivity of the ventral striatum predicted improvement in depressive and general psychiatric symptoms in the lithium group but only general psychiatric symptoms in the quetiapine group (Supplementary Figures 2–3). As noted in another study of this sample^46^, the findings favour lithium over quetiapine and point to a recurrent theme that implicates the ventral striatum as a major culprit in mediating manic symptoms and potentially the pharmacological effect of treatment.

The increased ventral striatal connectivity with the vmOFC and medial thalamus observed in patients at baseline is another indicator that the canonical ventral (limbic) corticostriatal circuit is compromised in mania. The vmOFC coordinates and integrates somatosensory information, including emotion, in decision-making and plays a role in assigning positive value to stimuli^56^. Bipolar patients and their unaffected first degree relatives illicit similar responses in the vmOFC even in the absence of rewarding stimuli, and have previously been shown to exhibit alterations in striatothalamic functional connectivity akin to their affected relatives^24,57^. These findings suggest that functional connectivity disturbances in ventral striatum connectivity could be a trait marker for the illness. This notion is further supported by the observation that increased connectivity of the ventral striatum was present at baseline in both treatment groups after the stabilization phase. In other words, the disturbance may not be related to the onset of the illness per se but to other biological factors closer to the etiology of the illness^58^.

Notably, FEM patients also showed reduced functional connectivity in the dorsal corticostriatal system at baseline. We have observed a similar gradient of dorsal-to-ventral, hypoconnectivity-to-hyperconnectivity in a separate sample of patients with first episode schizophrenia-like psychosis and their unaffected relatives^38^. We have also found hypoconnectivity of the dorsal system in people with an at-risk mental state for psychosis^59^. Most patients in this study had experienced an episode of psychosis, suggesting that dorsal corticostriatal hypoconnectivity may be a risk marker for psychosis that transcends traditional diagnostic boundaries. Further work examining how corticostriatal connectivity covaries with specific symptoms or syndromes across diagnoses will be required to test this hypothesis.

Strengths of the study include the relative homogeneity of the sample, the selection of a first episode cohort, randomized assignment to treatment groups and a longitudinal design. The study included 10% of individuals with schizoaffective disorder and substance-induced mania, which may have influenced the results, potentially favoring quetiapine given its efficacy in treating schizoaffective disorders^60^. Nonetheless, the number of subjects with either disorder was matched in the two treatment groups, thus reducing the variability contributed by these diagnoses and rendering the study more representative of FEM patient samples in Western societies^22,24^. Limitations include the relatively modest sample, which is expected for such a difficult to recruit and engage population. Multi-site investigations may prove useful in establishing the samples required for more robust multivariate methods to assess the utility of striatal functional connectivity in predicting treatment effects.

## Electronic supplementary material

Supplemental Materials

Supplemental Figure 1

Supplemental Figure 2

Supplemental Figure 3

Supplemental Figure 4
