# Supplementary material for: Differential effect of quetiapine and lithium on functional connectivity of the striatum in first episode mania
Source: Transl Psychiatry. 2018 Mar 6;8:59. doi: 10.1038/s41398-018-0108-8 (PMC5838223; doi:10.1038/s41398-018-0108-8)
Supplement: Supplementary file 1 — Supplemental Materials [file 41398_2018_108_MOESM1_ESM.doc]

Supplementary Materials

Figure 1: Z-score map of brain regions showing functional connectivity with the visual cortex seed.

Figure 2: Z-score map of brain regions demonstrating functional connectivity with the ventral striatum and correlation with change in symptoms scores after 12 months in the lithium group. Results are displayed at *P*<0.05 (FWE cluster corrected). Results remain significant when outliers are removed. For correlation values see Results section.

Figure 3: Z-score map of brain regions demonstrating functional connectivity with the ventral striatum and correlation with change in symptoms scores after 12 months in the quetiapine group. Results are displayed at *P*<0.05 (FWE cluster corrected). For correlation values see Results section.

Figure 4: Brain regions demonstrating significant increase in functional connectivity with the ventral striatum in FEM patients compared to healthy control subjects at baseline. The slices are viewed in MNI standard space.
