# Supplementary figures and images for: Differential effect of quetiapine and lithium on functional connectivity of the striatum in first episode mania
Source: Transl Psychiatry. 2018 Mar 6;8:59. doi: 10.1038/s41398-018-0108-8 (PMC5838223; doi:10.1038/s41398-018-0108-8)

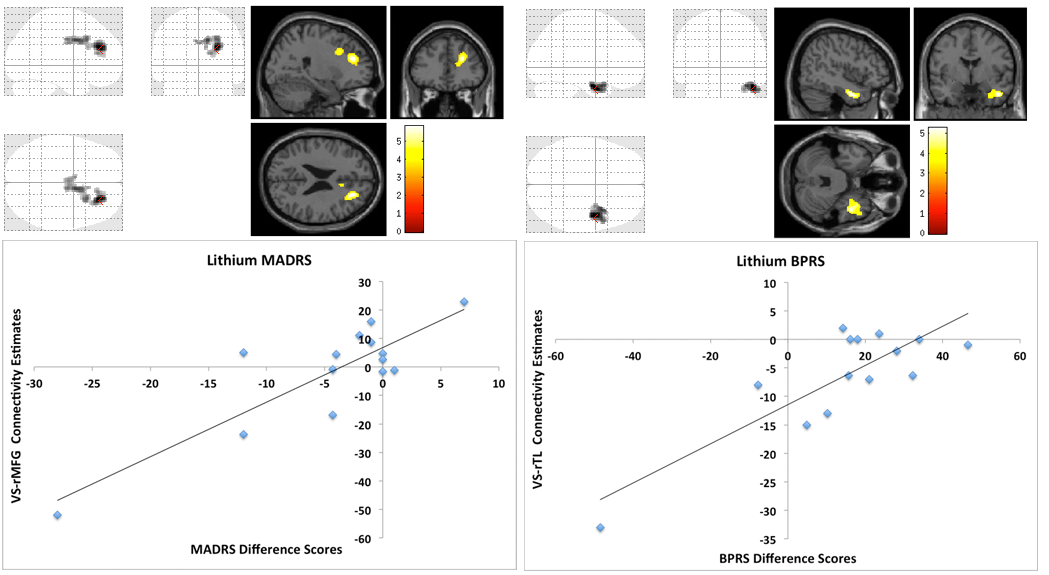

Supplement: Supplementary file 3 — Supplemental Figure 2 [file 41398_2018_108_MOESM3_ESM.tif]

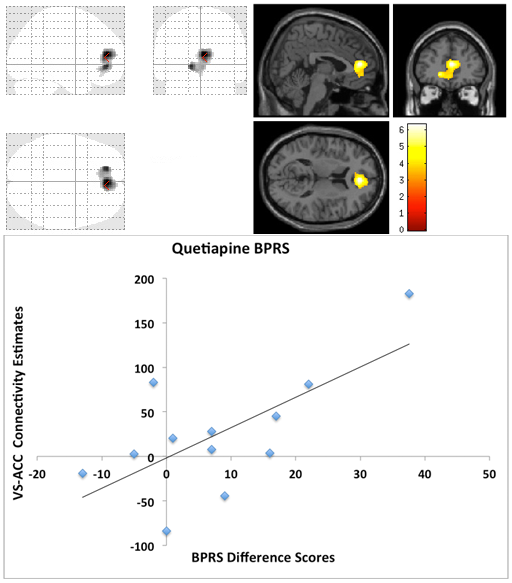

Supplement: Supplementary file 4 — Supplemental Figure 3 [file 41398_2018_108_MOESM4_ESM.tif]

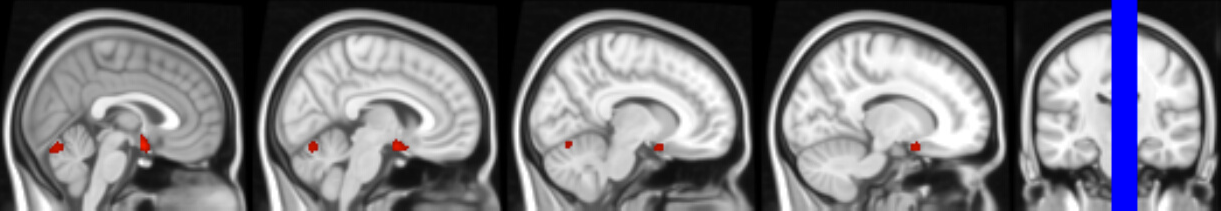

Supplement: Supplementary file 5 — Supplemental Figure 4 [file 41398_2018_108_MOESM5_ESM.tif]
